# Biochemical algorithm to identify individuals with *ALPL* variants among subjects with persistent hypophosphatasaemia

**DOI:** 10.1186/s13023-022-02253-5

**Published:** 2022-03-03

**Authors:** C. Tornero, V. Navarro-Compán, A. Buño, K. E. Heath, M. Díaz-Almirón, A. Balsa, J. A. Tenorio, J. Quer, P. Aguado

**Affiliations:** 1 Department of Rheumatology, La Paz University Hospital, IdiPaz, Paseo de La Castellana, 261, 28046 Madrid, Spain; 2 Skeletal Dysplasia Multidisciplinary Unit (UMDE) and ERN-BOND, La Paz University Hospital, Madrid, Spain; 3 Department of Clinical Biochemistry, La Paz University Hospital, Madrid, Spain; 4 Institute of Medical and Molecular Genetics (INGEMM), La Paz University Hospital, IdiPAZ, Universidad Autónoma de Madrid, Madrid, Spain; 5 CIBERER (Centro de Investigación Biomédica en Red de Enfermedades Raras), ISCIII, Madrid, Spain; 6 Department of Biostatistics, La Paz University Hospital, Madrid, Spain; 7 Masters in Telecommunications and Big Data, Telecommunications Engineering Degree, ICAI, Madrid, Spain

**Keywords:** Hypophosphatasia, Alkaline phosphatase, *ALPL*, Hypophosphatasaemia, Metabolic bone diseases

## Abstract

**Background:**

Hypophosphatasia (HPP) is a rare and underdiagnosed condition characterized by deficient bone and teeth mineralization. The aim of this study was first, to evaluate the diagnostic utility of employing alkaline phosphatase (ALP) threshold levels to identify adults with variants in *ALPL* among individuals with persistently low ALP levels and second, to determine the value of also including its substrates (serum pyridoxal-5′-phosphate—PLP—and urinary phosphoetanolamine-PEA) for this purpose in order to create a biochemical algorithm that could facilitate the diagnostic work-up of HPP.

**Results:**

The study population comprised 77 subjects with persistent hypophosphatasaemia. They were divided into two groups according to the presence (+GT) or absence (−GT) of pathogenic *ALPL* variants: 40 +GT and 37 −GT. Diagnostic utility measures were calculated for different ALP thresholds and Receiver Operating Characteristic (ROC) curves were employed to determine PLP and PEA optimal cut-off levels to predict the presence of variants. The optimal threshold for ALP was 25 IU/L; for PLP, 180 nmol/L and for PEA, 30 µmol/g creatinine. Biochemical predictive models were assessed using binary logistic regression analysis and bootstrapping machine learning technique and results were then validated. For ALP < 25 UI/L (model 1), the area under curve (AUC) and the 95% confidence intervals (CI) was 0.68 (95% CI 0.63–0.72) and it improved to 0.87 (95% CI 0.8–0.9), when PEA or PLP threshold levels were added (models 2 and 3), reaching 0.94 (0.91–0.97) when both substrates were included (model 4). The internal validation showed that the addition of serum PLP threshold levels to the model just including ALP improved significantly sensitivity (S) and negative predictive value (NPV) − 100%, respectively- with an accuracy (AC) of 93% in comparison to the inclusion of urinary PEA (S: 71%; NPV 75% and AC: 79%) and similar diagnostic utility measures as those observed in model 3 were detected when both substrates were added.

**Conclusions:**

In this study, we propose a biochemical predictive model based on the threshold levels of the main biochemical markers of HPP (ALP < 25 IU/L and PLP > 180 nmol/L) that when combined, seem to be very useful to identify individuals with *ALPL* variants.

**Supplementary Information:**

The online version contains supplementary material available at 10.1186/s13023-022-02253-5.

## Background

Hypophosphatasia (HPP) is a rare inborn-error-of-metabolism characterized by low activity of the tissue non-specific isoenzyme of alkaline phosphatase (TNSALP). This deficiency in TNSALP activity leads to extracellular accumulation of its natural substrates: inorganic pyrophosphate (PPi), pyridoxal-5′-phosphate (PLP) and phosphoethanolamine (PEA). PPi inhibits hydroxyapatite crystal growth, impairing bone and tooth mineralization, which could result in rickets in infants and children, osteomalacia in adults, recurrent fractures, premature loss of deciduous teeth and dental abnormalities, chondrocalcinosis, calcific periarthritis, musculoskeletal pain or muscle weakness among others [1, 2]. Seven principal subtypes of HPP are historically recognized: perinatal, infantile, childhood, adult, odontohypophosphatasia, perinatal benign and the extremely rare, pseudohypophosphatasia [3] Nevertheless, the separation among these subtypes is not well established and overlapping symptomatology or severe complications may manifest at any age [4, 5] The main biochemical hallmark of the disease is hypophosphatasaemia; however, in clinical practice, low ALP levels are often overlooked and their causes are not usually investigated [6]. Persistent hypophosphatasaemia can also occur due to the use of certain drugs (such as bone antiresorptives, corticosteroids, chemotherapy or clofibrate), in cases of certain diseases (profound hypothyroidism or anaemia, Cushing syndrome, Wilson disease or cancer), vitamin deficiencies, acute processes (cardiac bypass surgery, massive transfusions, etc.) and as well as in other metabolic disorders, which must be considered when evaluating patients with this biochemical abnormality [7].

In order to facilitate the diagnosis of HPP in adults, algorithms combining suggestive clinical symptoms and the presence of low ALP levels and substrates accumulation have been proposed [8]. Although not considered necessary for the diagnosis, genetic analysis is a useful method for supporting it and it also provides information about inheritance patterns and for genetic counseling [9]. In clinical practice, on the other hand, access to a genetic assessment is not always possible and can even delay early diagnosis.

In this context, we tried to determine those clinical and biochemical characteristics that might better identify those patients with *ALPL* variants. In a previous cross-sectional study of our group [10], subjects with persistent hypophosphatasaemia and a positive *ALPL* genetic test were compared with another group of individuals with the same biochemical abnormality and a negative genetic result. We found that musculoskeletal pain and ALP levels < 25 IU/L were associated with the presence of *ALPL* variants. This threshold showed a specificity, positive predictive value and positive likelihood ratio of 97.8, 94.4% and 19.8 to detect a positive *ALPL* genetic test, respectively. As cross-sectional studies have methodological limitations, especially those evaluating biochemical parameters that could be susceptible to variations, we conducted this longitudinal study in order to obtain more consistent and reliable data. In this context, the aim of this study was first, to evaluate the diagnostic utility of employing alkaline phosphatase (ALP) threshold levels to identify adults with variants in *ALPL* among individuals with persistently low ALP levels and second, to determine the value of also including TNSALP substrates (serum pyridoxal-5′-phosphate-PLP- and urinary phosphoetanolamine-PEA) for this purpose in order to facilitate a diagnostic work-up of the disease.

## Results

### Demographics and clinical, biochemical and genetic assessments

The population for this study consisted of 77 adult subjects with persistent hypophosphatasaemia (≥ 2 ALP values ≤ 35 IU/L and none > 45 IU/L), of whom 40 (51.9%) had single heterozygous disease-causing variants in *ALPL* (+GT) while 37 (48.1%) did not (−GT). Demographics and clinical characteristics of study participants at baseline are shown in Table 1. Compared with the −GT group, the +GT group included a lower percentage of females and had a higher BMI. There were no significant differences in age. Patients in the +GT group presented more musculoskeletal pain and dental abnormalities compared to the −GT group (p < 0.05).

**Table 1:** Demographics and clinical baseline characteristics of the two subgroups

	+GT (n = 40)	−GT (n = 37)	p value
Caucasian race, n (%)	39 (97.5)	37 (100%)	0.8
Median age (IQR), years	52.8 (38.8–61.2)	46.6 (40.1–49.9)	0.2
Female sex, n (%)	27 (67.5%)	32 (86.8%)	0.05
Median BMI (IQR), kg/m^2^	25.2 (23–29)	22.6 (20.6–24.6)	< 0.01
Musculoskeletal pain, n (%)	28 (70%)	18 (48.6%)	< 0.05
Dental abnormalities, n (%)	16 (40%)	4 (11%)	< 0.001
Premature teeth loss or multiple extractions, n (%)	3 (7.5%)	1 (2.7%)	0.3
Calcific periarthritis, n (%)	7 (18%)	4 (10.8%)	0.4
PH of fractures, n (%)	16 (40%)	14 (37.8%)	0.5
PH of stress fractures, n (%)	6 (15%)	1 (2.7%)	0.06

Significant values are shown in bold

+*GT* positive genetic test, −GT negative genetic test, *PH* personal history

**Fig. 1:**
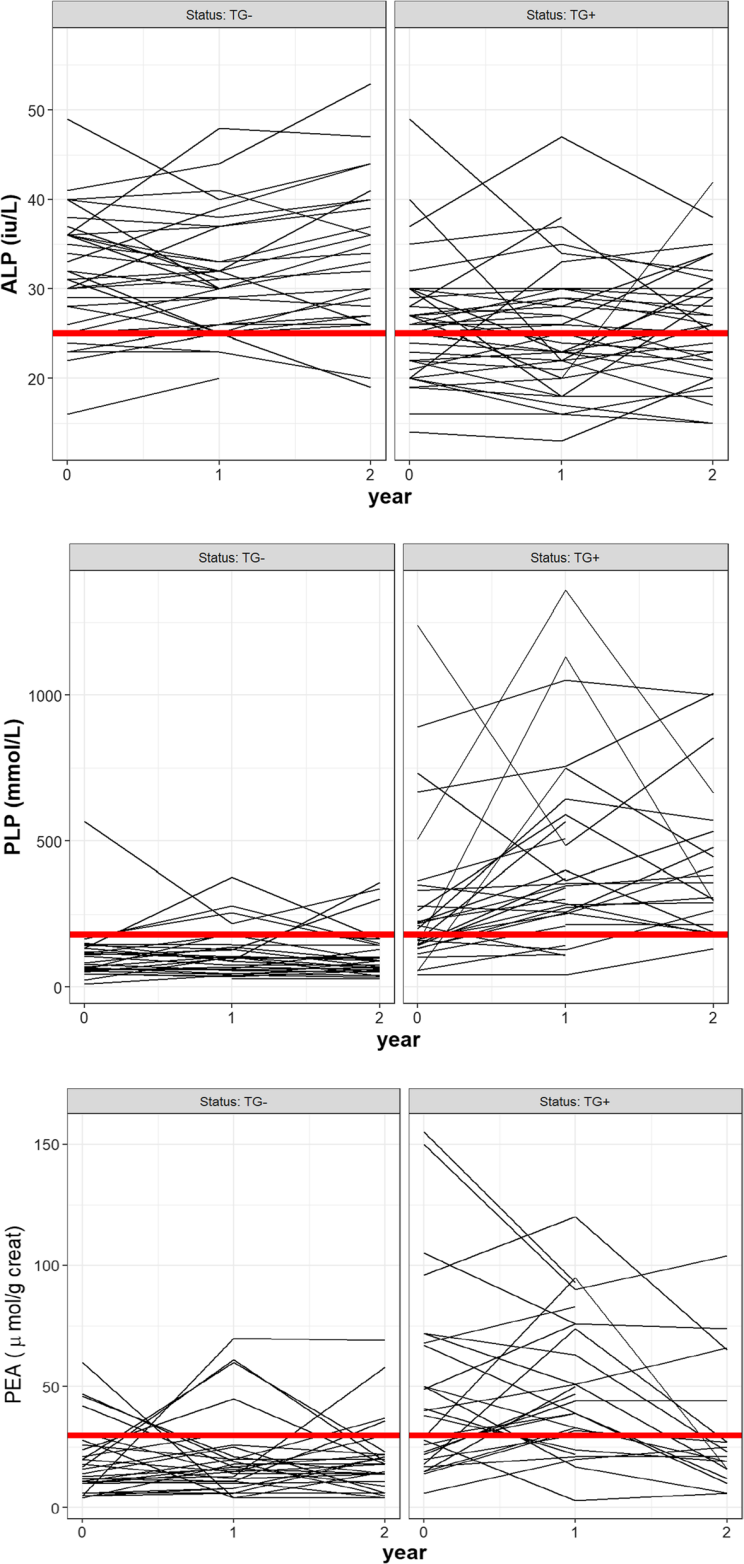
Individual ALP and substrates measurements at baseline, 1- and 2-year visits. Abbreviations: *ALP* alkaline phosphatase (IU/L), *PLP* serum pyridoxal-5′-phosphate (nmol/L), *PEA* urinary phosphoetanolamine (µmol/g creatinine). Red lines represent the proposed threshold levels for ALP (< 25 IU/L) and substrates (PLP > 180 nmol/L and PEA > 30 µmol/g creatinine)

In terms of the biochemical assessment at baseline, ALP levels (IU/L) were significantly lower and substrates (PLP and PEA), consistently higher, in the +GT group in comparison to the other group and these differences persisted during all the visits (p < 0.001). Table 2 details biochemical measurements stratified by groups for all the visits. Data relating to reference biological intervals in adults *(*for ALP, 45–116 IU/L; for PLP, 15–73 nmol/L and for PEA, below 70 µmol/g creatinine), instrument and methods of analysis are included in the Methods section. Correlated data were adjusted to Generalized Linear Mixed Models (GLMs) and three GLMs were performed in order to assess the interaction of time and genetic status (time*GS) effect. For each model (with a binary yes/no response for ALP < 25 IU/L, PLP > 180 nmol/L and PEA > 30 µmol/g.creatinine), time, genetic status and time*GS were evaluated. The results revealed that time*GS did not affect the probability of these binary responses (for ALP < 25 IU/L response, *p* = 0.39; for PLP > 180 nmol/L, *p* = 0.66 and for PEA > 30 µmol/g.creatinine, *p* = 0.27). Hence, as time trends per group were not observed, median levels from all the visits were used for the development of the biochemical predictive models. The evolution of individual measurements of these biochemical parameters are included in Fig. 1 and the probabilities of the binary responses for ALP and the substrates' proposed threshold levels are presented in Additional file 1: Table S1.

**Table 2:** Evolution of serum alkaline phosphatase, serum piridoxal-5-phosphate and urinary phosphoetanolamine levels in both groups

	Baseline		6 months		1 year		18 months		2 years	
	−GT	+GT	−GT	+GT	−GT	+GT	−GT	+GT	−GT	+GT
ALP, IU/L	31 (27–36)	26 (22–30)*	30 (26–35.8)	25 (20.5–27.5*	30.5 (26–37)	24.5 (21–29)*	31 (27–40)	25 (21.8–29)*	33 (27–40)	26 (20.75–30.25)*
PLP (nmol/L)	79.49 (57.7–124.4)	200 (132.75–270.9)*			99.54 (52.2–141.92)	345.35 (242.67–573.28)*			82.26 (56.18–143.49)	293.9 (207.5–479.2)*
PEA µmol/g. creatinine	13.5 (8.25–26.5)	40.5 (18.5–67.8)*			16 (11–22)	41.5 (25.5–75.5)*			18 (12.5–24)	22 (16–44)*

Data are expressed as median (interquartile range); *p value < 0.001 in the comparison of biochemical parameters between the +GT and −GT groups during the visits assessed

+*GT* positive genetic test, −GT negative genetic test, *Bsl* baseline, *m* months, *y* years, *ALP* alkaline phosphatase

Regarding subjects who had variants in *ALPL*, 35 (87.5%) were heterozygous for pathogenic variants and 5 (12.5%), for a likely pathogenic variant. The majority of the variants were localized in exons 5, 6 and 9, were predicted to have a damaging effect based on silico pathogenicity prediction tools and were absent or present at extremely low frequencies in gnomAD. The most frequent variant was p.(Thr115_Ala116dup), was observed in 5 patients; p.(Glu291Lys) was observed in 3 patients and p.(Gly112Arg), p.(Thr166Ile), p.(Asp378Gly), p.(Gly491Arg), p.(Glu291Lys) and c.473-2A > G in 2 patients each. The remaining variants were observed in single subjects. Additional file 2: Table S2 includes the genetic assessments of the subjects in the +GT group.

### Logistic regression models and bootstrapping resampling

First, in order to confirm the threshold of ALP levels < 25 IU/L observed in our transversal study as a good predictor of *ALPL* variants, diagnostic utility measures were computed for all of the visits. Sensitivity (S) ranged between 37.5 and 48.7%; specificity (Sp), from 83.8 to 92.6%; positive predictive value (PPV), between 71.4 to 87.5% and negative predictive value (NPV) from 55.4 to 60.8% (see Table 3 for further details). Second, Receiver Operating Characteristic (ROC) curves were employed to determine PLP and PEA best cut-off levels to predict *ALPL* disease-causing variants and revealed that the threshold levels with the optimal combination of sensitivity and specificity were 180 nmol/L for PLP (S: 76%, Sp: 95%) and 30 µmol/g creatinine (S: 56%, Sp: 97%) for PEA.

**Table 3:** Diagnostic utility measures for different ALP thresholds during the two-year follow up evolution

ALP levels (IU/L)	S (%)	Sp (%)	PPV (%)	NPV (%)	+LR	−LR
*Baseline*						
< 20	10.0	97.3	80.0	50.0	3.70	0.92
< 25	37.5	83.8	71.4	55.4	2.31	0.75
< 30	72.5	64.9	69.0	68.6	2.07	0.42
< 35	90.0	37.8	61.0	77.8	1.45	0.26
*6-month visit*						
< 20	18.2	96.4	85.7	50.0	5.06	0.85
< 25	48.5	85.7	80.0	58.5	3.39	0.60
< 30	78.8	50.0	65.0	66.7	1.58	0.42
< 35	87.9	35.7	61.7	71.4	1.37	0.34
*One-year visit*						
< 20	17.9	100	100	51.5	∞	0.82
< 25	48.7	91.2	86.4	60.8	5.53	0.56
< 30	76.9	61.8	69.8	70.0	2.01	0.37
< 35	89.7	26.5	58.3	69.2	1.22	0.39
*18-month visit*						
< 20	14.7	96.3	83.3	47.3	3.97	0.89
< 25	47.1	89.0	84.2	57.1	4.24	0.60
< 30	82.4	55.6	70.0	71.4	1.86	0.32
< 35	91.2	37.0	64.6	76.9	1.45	0.24
*Two-year visit*						
< 20	17.4	96.3	83.3	47.3	3.97	0.89
< 25	41.2	92.6	87.5	55.6	5.57	0.63
< 30	70.6	66.7	72.7	64.3	2.12	0.44
< 35	91.2	44.4	67.4	80.0	1.64	0.20

*ALP* alkaline phosphatase (IU/L), *S* sensitivity, *Sp* specificity, *PPV* positive predictive value, *NPV* negative predictive value, *LR* likelihood ratio

Posteriorly, in the training set, binary logistic regression (BLR) models applying the above mentioned ALP, PLP and PEA threshold levels (25 IU/L for ALP, 180 nmol/L for PLP and 30 µmol/g creatinine) were developed (Table 4) and bootstrapping machine learning analysis of 500 resames was performed (Table 5) to calculate the area under the curve (AUC) and the 95% confidence interval (CI). For ALP < 25 UI/L (model 1), the AUC was 0.68 (95% CI 0.63–0.72). It improved to 0.87 (95% CI 0.82–0.9) when PEA or PLP threshold levels were added (models 2 and 3) and it reached 0.94 (0.91–0.97) when both substrates were included (model 4). Finally, the validation of the models was performed in 20% of the study population (Table 4). Model 1, including ALP levels < 25 UI/l showed S: 57%; Sp: 85%; PVV; 80%; NPV: 66%; +LR: 3.99; −LR: 0.5 and an accuracy of 71%. When ALP levels < 25 IU/L were combined with PLP > 180 nmol/L (model 3), diagnostic utility measures improved significantly (S: 100%; Sp: 86%; PVV: 88%; NPV: 100%; +LR: 6.99; −LR: 0 with an accuracy of 93%) in comparison to the inclusion of urinary PEA in model 2 (S: 71%; NPV 75% and AC: 79%) and were similar to those including both substrates (model 4). For model 5, significant clinical features in the univariate analysis were added to ALP and substrates, but failed to improve the model (see Table 4 for further details).

**Table 4:** Logistic regression models and diagnostic utility measures to identify individuals with *ALPL* variants

	Variables included	B	Odds ratio (95% CI)	P value	Diagnostic utility measures
Model 1:	ALP < 25	ALP: − 1.62	0.2 (0.05–0.54)	< 0.01	S: 57%; Sp: 85%; PPV: 80%; NPV: 66%; +LR: 3.99; −LR:0.5; AC: 71%
		C: − 0.72	0.48 (0.18–0.99)	0.06	
Model 2:	ALP < 25 + PEA > 30	ALP: − 1.82	0.16 (0.04–0.54)	< 0.01	S: 71%; Sp: 86%; PPV: 83%; NPV: 75%; +LR: 4.99; −LR: 0.33; AC: 79%
		PEA: − 2.43	0.09 (0.02–0.27)	< 0.001	
		C: − 1.42	0.24 (0.07–0.63)	< 0.01	
Model 3:	ALP < 25 + PLP > 180	ALP: − 1.38	0.25 (0.05–0.93)	0.02	S: 100%; Sp: 86%; PPV:88%; NPV: 100%; +LR: 6.99; −LR:0; AC: 93%
		PLP: − 2.81	0.06 (0.01–0.22)	0.04	
		C: − 1.51	0.22 (0.04–0.65)	< 0.001	
Model 4:	ALP < 25 + PLP > 180 + PEA > 30	ALP: − 2.3	0.1 (0.01–0.29)	0.02	S: 100%; Sp: 86%; PPV:88%; NPV: 100%; + LR: 6.99; −LR:0; AC: 93%
		PLP: − 3.30	0.04 (0.01–0.2)	< 0.01	
		PEA: − 2.94	0.05 (0.01–0.25)	< 0.01	
		C: − 2.83	0.06 (0.01–0.29)	< 0.01	
Model 5:	ALP < 25 + PLP > 180 + PEA > 30 + C. Pain + Dental ab	ALP: − 2.27		0.02	
		PLP: − 3.72		< 0.01	
		PEA: − 3.82		< 0.02	
		CP:2.34		0.16	
		DA: − 0.76		0.55	
		C: − 3.27			

*ALP* alkaline phosphatase (IU/L), *PLP* serum pyridoxal-5′-phosphate (nmol/L), *PEA* urinary phosphoetanolamine (µmol/g creatinine), *C. pain* chronic pain, *Dental ab* dental abnormalities, *C* constant, *S* sensitivity, *Sp* specificity, *PPV* positive predictive value, *NPV* negative predictive value, *LR* likelihood ratio, *AC* accuracy

**Table 5:** Bootstrapping method descriptive results of the 500 resamples for the different biochemical models assessed

	Variables included	AUC ROC curve
Model 1	ALP < 25	0.68 (0.63–0.72)
Model 2	ALP < 25 + PEA > 30	0.87 (0.82–0.9)
Model 3	ALP < 25 + PLP > 180	0.87 (0.82–0.91)
Model 4	ALP < 25 + PLP > 180 + PEA > 30	0.94 (0.91–0.97)

*ALP* alkaline phosphatase (IU/L), *PLP* serum pyridoxal-5′-phosphate (nmol/L), *PEA* urinary phosphoetanolamine (µmol/g creatinine), *AUC* area under the curve, *ROC curve* receiver operating characteristic curve

## Discussion

This study confirms that ALP levels < 25 IU/L could be useful for identifying individuals harboring *ALPL* variants and reveals that its combination with the proposed substrate cut-off levels improve diagnostic utility measures and predict, with a high accuracy, the presence of *ALPL* variants. In the field of rare diseases, characterized by a significant delay in diagnosis, we propose a biochemical predictive model of reduced complexity that could help physicians in the diagnostic work-up of patients with clinical suspicion of the disease.

The clinical spectrum and severity of HPP is variable and different *ALPL* gene variants and inheritance patterns can greatly affect the clinical expression of the disease in adults [11]. As low ALP levels are frequently overlooked [6], HPP is frequently unrecognized, with important consequences, such as erroneous diagnosis or the use of contraindicated medications [12, 13]. In clinical practice, on the other hand, when hypophosphatasaemia is identified a diagnostic work-up is still needed to diagnose HPP. In terms of its clinical features, previous studies comparing subjects with persistently low ALP levels displaying *ALPL* variants (or not) found either mild ailments in patients with a positive genetic study [14] or no statistically significant clinical differences in clinical symptoms [15]. By contrast, in our study we found more chronic musculoskeletal pain and dental abnormalities in patients with *ALPL* variants. Biochemically, we also observed differences between groups, with lower ALP levels and higher substrates in subjects with mutations, in agreement with the results observed in other cohorts [14].

In a previous cross-sectional study [10], we found that musculoskeletal pain and ALP levels < 25 IU/L associated with a positive genetic results and these threshold levels seemed to be useful for predicting the presence of mutations. These results have been confirmed in this longitudinal study and, additionally, BLR and machine learning techniques have shown that including substrates in the algorithm more accurately predicted the presence of mutations. The internal validation of the BLR model revealed the following diagnostic utility measures for model 1 (ALP levels < 25 IU/L): S: 57%; Sp: 85%; PPV: 80%; NPV: 66% and AC: 71%. The addition of serum PLP threshold levels to this model (model 3), improved significantly S and NVP (100%, respectively) with an AC of 93% in comparison to the inclusion of urinary PEA (model 2) (S: 71%; NPV 75% and AC: 79%). Similar diagnostic utility measures as those observed in model 3 were detected when both substrates were added (model 4). These results are in agreement with previous studies: although both serum PLP and urinary PEA are useful for the diagnosis of HPP, the former seems to have a higher sensitivity [2, 14, 16, 17] and in addition, both in paediatric and adult patients, higher levels seem to reflect a more severe disease [18, 19]. By contrast, PEA is not pathognomonic, can be unremarkable in mild HPP and their excretion may be influenced by age, diet or circadian rhythm so consequently, it is considered of secondary importance [20, 21]. Besides, the proposed PEA threshold was still within the normal range (< 70 µmol/g creatinine).

Bootstrapping machine learning analysis was employed to compare the AUC (95% CI) of the predictive models: model 1 (ALP levels < 25 IU/L), presented an AUC of 0.68 (95% CI 0.6–0.7). It improved when only one of the substrates (models 2 and 3) was added to the model, with an AUC of 0.87 (95% CI 0.8–0.9), reaching 0.94 (95% CI 0.91–0.97) when both ALP and substrates conformed the model (model 4). Although model 4 could appear the most optimal one, our results support that, for the above-mentioned reasons, the combination of ALP levels < 25 IU/L and PLP levels > 180 nmol/L (model 3) seems to be the most cost-efficient model, especially when access to substrate quantitation is limited. In a retrospective transversal study, Garcia-Carretero et al. [22] also explored the possibility of predicting *ALPL* mutations using biochemical algorithms. ALP threshold levels below or equal to 24 UI/L, or ALP levels between 24 and 32 IU/L combined with PLP levels above 45 ng/ml (equivalent to 156 nmol/L) were associated to a high suspicion of HPP. In our prospective study, similar ALP and PLP threshold levels have been proposed as good predictors of detecting variants. In contrast to this study, we observed clinical features differentiating both groups, although when clinical symptoms were added to our biochemical predictive model, they did not improve it. In this sense, it is worth noting that the use of this biochemical algorithm is proposed to help to reduce uncertainty in the diagnostic work-up and management of the disease in patients with a previous clinical suspicion and that the clinical perspective cannot be forgotten: biochemical abnormalities become important when accompanied by the presence of symptoms characterizing the disease.

Among the main strengths to be highlighted of this study is the longitudinal prospective design which has allowed us to create a biochemical algorithm based on more consistent data. Moreover, its design ensures as much as possible, the absence of secondary causes of persistently low ALP levels and/or the effects of multivitamin supplements, which could distort biochemical measurements. From the statistical point of view, it is noteworthy the use of machine learning techniques complementing standard statistical methods. However, the study limitations include its small sample size, although significant for a rare disease, the strict selection criteria and the requirement for further external validation in larger cohorts. In the field of prediction, external validation is required to create appropriate trust in a model before it can be extrapolated to other populations [23]. For these reasons, our results are exploratory and should be interpreted cautiously. Disease registers, multicenter collaborations and machine learning with access to big data could be of interest to determine the generalizability of our model to new and different patients.

## Conclusions

In summary, we propose a biochemical predictive model based on the threshold levels of the main biochemical markers of the disease (ALP levels < 25 IU/L and PLP levels > 180 nmol/L) that when combined, seem to be very useful to identify individuals with *ALPL* variants. This is a very interesting avenue and requires further exploration with external data sets. We are confident these results, if confirmed in other cohorts, could be very useful in the diagnostic work-up of HPP.

## Methods

### Study population and design

Two-year longitudinal prospective study performed at La Paz University Hospital in Madrid (Spain) from September 2018 to September 2020. This study was approved by the Ethics Committee of La Paz University Hospital and written informed consent was obtained from all of the subjects.

Patients recruited in a previous cross-sectional study performed at our Hospital in 2016 were offered the opportunity to participate in this longitudinal study, as also were those patients later diagnosed with HPP afterwards at our clinical practice before September 2018. Details related to the recruitment process for this cross-sectional study were reported in a previous publication by our group [10]. In summary, 1,536,711 laboratory records of ALP serum levels from 386,353 subjects recorded in the biochemical database of our hospital between 2009 and 2015 were screened to identify persistently low ALP results. Of these patients, 231,805 were adults with at least two ALP measurements, of whom 427 presented persistent hypophosphatasaemia (≥ 2 ALP values ≤ 35 IU/L and none > 45 IU/L). Thirty-one subjects were excluded because of secondary causes of low ALP levels [7] and 13 because they could not be contacted by telephone. A total of 383 adults presented ≥ 2 ALP measurements below or equal to 35 IU/L and none > 45 were contacted by telephone. Eighty-five decided to participate in the cross-sectional study and signed the informed consent for genetic testing, of whom 39 (46%) patients presented pathogenic (P) or likely pathogenic (LP) disease-causing variants.

**Fig. 2:**
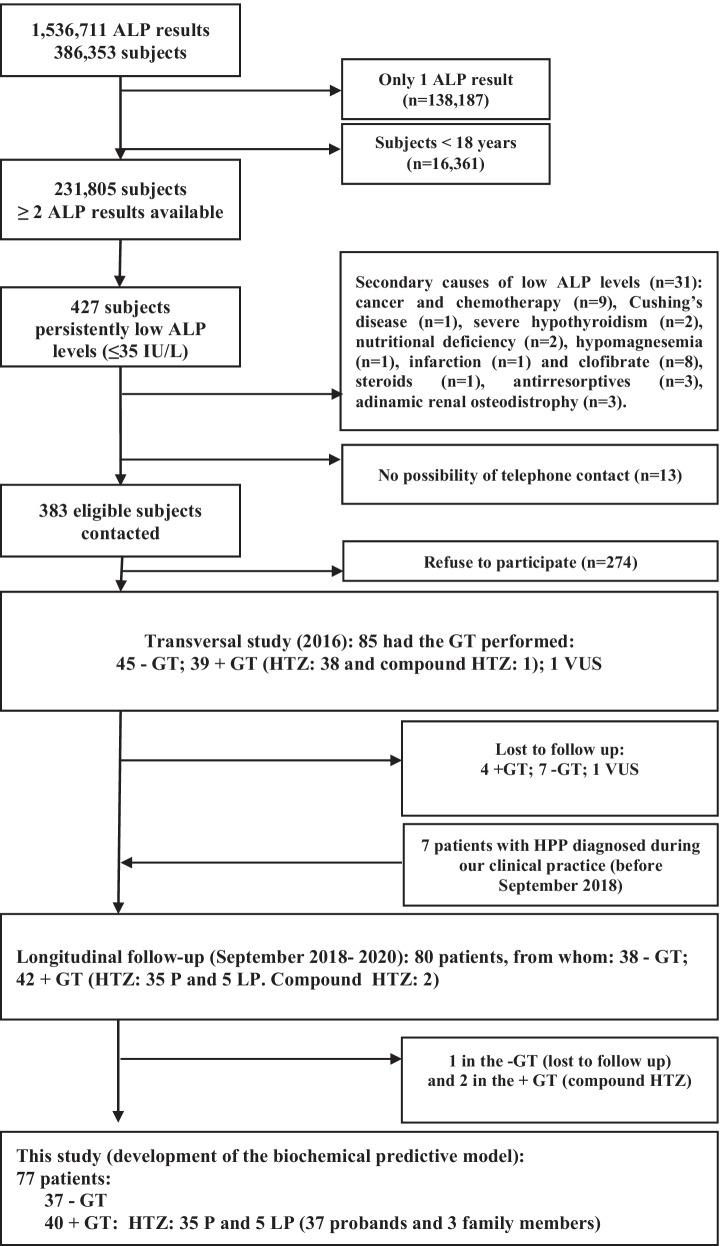
Flowchart describing screening and selection process. Abbreviations: *ALP* alkaline phosphatase (IU/L), +*GT* positive genetic test, −*GT* negative genetic test, *VUS* variant of unknown significance, *P* pathogenic variant, *LP* likely pathogenic variant, *HTZs* heterozygotes

These subjects were invited to participate in a longitudinal prospective study as also were seven patients later diagnosed of HPP before September 2018 in our Rheumatology Department. The main criteria for inclusion were: adults aged 18 years or older with persistent hypophosphatasaemia, defined as ≥ 2 ALP measurements below or equal to 35 IU/L, none > 45 IU/L and with a genetic *ALPL* screening performed at our Hospital. Eighty patients (42 patients with a positive HPP genetic test and 38 with a negative genetic analysis) agreed to participate in the longitudinal two-year follow-up study. Nevertheless, for the present study, in order to develop the biochemical predictive algorithm, the two patients in the +GT group who were compound heterozygotes for *ALPL* variants were excluded from the analysis -to obtain a more homogeneous population- and one in the −GT group, because of lost to follow-up. Thus, the population included in this study consisted of 77 adult subjects with persistent hypophosphatasaemia: 40 (51.9%) with heterozygous disease-causing variants in *ALPL* and 37 (48.1%) without mutations. Flowchart describing screening and selection process is included in Fig. 2.

### Clinical symptoms and laboratory methods

A personal history of musculoskeletal pain, fractures, dental abnormalities or premature teeth loss and calcific periarthritis among other clinical features classically associated with HPP, were evaluated during our consultations. In order to evaluate whether certain clinical features could improve our biochemical predictive model, their values at baseline were employed. Musculoskeletal pain was considered present when symptoms were recurring or chronic (> 6 months) and not when transient. Dental abnormalities were defined as tooth shape abnormalities, structure and colour abnormalities of the enamel or dentin, thin enamel, late teeth eruption or severe/recurrent cavities and early loss of permanent teeth was defined as the loss of several teeth or extractions (> 10) due to past tooth abnormalities, prior to age 50. In terms of fractures, stress metatarsal and atypical fractures were considered.

Biochemical parameters of the subjects included in this two-year prospective longitudinal study conducted at La Paz Hospital were utilized. ALP levels were evaluated every six months and TNSALP substrates were determined at baseline and then yearly. Between 2009 and 2013, the University La Paz Hospital Laboratory utilized an Olympus 5400 analyzer (Beckman Coulter) to measure serum ALP activity. In February 2014, it switched to Siemens Healthineers (Advia 2400 chemistry system). The Clinical Laboratory Service of our hospital has its technical competence accredited with the ISO 15189:2013 standard and the measurement of ALP activity is within this scope. Based on this, analytical correlation studies were performed (internal data) using serum samples from adults demonstrating interchangeability between both analyzers so the reference biological intervals (normal range) were the same for both of them. Both methods measure ALP activity by a kinetic rate method in which p-nitrophenyl phosphate (a colourless organic phosphate ester substrate) was hydrolyzed by ALP to the yellow-coloured product pnitrophenol and phosphate at pH 10.3. The enzymatic activity of ALP was directly proportional to changes in absorbance at 410 nm. A commercial kit was employed for its measurement in our laboratory and the normal adult's range was 45 to 116 IU/L according to the reference interval limits given by the manufacturer in the instructions for use (IFU) document. For PLP, an HPLC method using a commercial kit from Chomsystems (Teknokroma) was employed. PEA measurement was carried out using an in-house liquid chromatography/tandem mass spectrometry (LC–MS/MS) method adapted for quantifying aminoacids without derivatization. The normal adult’s range is 15–73 nmol/L for PLP and < 70 µmol/g creatinine for PEA, according to intervals recommended for adults by our reference laboratory that performed the analysis. Patients were required to avoid multi-supplement vitamin intake at least for one week before the analysis in order to avoid any interference with the TNSALP substrate measurements and to fast overnight.

### Genetic analysis

In all of the patients included, genomic DNA was extracted from peripheral blood with a Chemagic Blood kit (Perkin Elmer, Waltham, MA) and the screening of the exons and intron/exon boundaries of *ALPL* (NM_000478.6) was performed by Sanger sequencing. The allelic frequencies were determined using gnomAD (http://gnomad.broadinstitute.org/) and 11 in silico tools were employed to assess the pathogenicity, including CADD V1.4 (http://cadd.gs.washington.edu), DANN, SIFT, MutationTester, Mutation assessor and FATHM all available in Varsome (https://varsome.com/; Saphetor, Lausanne, Switzerland) and Polyphen and four splicing tools; SpliceSiteFinder-like, MaxEntScan, NNSPLICE, GeneSplicer, all available in Alamut V2.11 software (Interactive Biosoftware, Rouen, France); along with the Silvent et al. criteria [24]. The *ALPL *disease-causing variants database (http://www.se-sep.uvsq.fr/03_hypo_mutations.php) was also consulted to obtain up-to-date information about the genetic variants included in our study. They were classified according to the American College of Medical Genetics and Genomics (ACMG) standards and guidelines [25].

### Statistical analysis

Descriptive analysis was employed to compare clinical and biochemical features among individuals with and without *ALPL* variants. Continuous variables were described as median (interquartile range − IQR-) and categorical variables as an absolute number and relative percentage. At baseline, comparisons between two independent groups for continuous variables were performed using the Student’s *t*-test for unpaired data if normally distributed, or the Mann–Whitney U-test when not. The Chi-square test was used for categorical variables.

In order to confirm ALP cut-off levels < 25 IU/L proposed in a previous cross-sectional study of our group [10] as a good predictor of the presence of *ALPL* disease-causing variants, diagnostic utility measures were calculated for all of the visits and ROC curves were employed to determine PLP and PEA optimal cut-off levels for this purpose. GLMs were performed in order to ensure that the interaction of time and genetic status did not affect median ALP and substrates' measurements. Posteriorly, biochemical predictive models based on the combination of ALP and substrate's threshold levels were created. For their assessment, the total sample was partitioned into training (80%) and validation (20%) groups. In the training set, BLR analysis was employed. This is an statistical method that allows to estimate the probability of a binary outcome and, in this study, it was used to describe the relative contribution of each independent variable to the outcome by controlling for the influences of the other independent variables [26] allowing us to develop the biochemical predictive models. Additionally, bootstrapping, a machine learning technique, which involves taking random samples from the dataset with re-selection of 500 resamples (*B* = 500) was used to compare different models employing the area under the curve (AUC) and the 95% confidence interval [27]. Finally, their validation was performed in the corresponding set and diagnostic utility measures were calculated for all of them. The level of statistical significance was set at *p* < 0.05. To perform the analysis, we used R Studio v.1.2.5042 © 2009-2020 Inc. The package used to perform the graphs was ggplot2 v3.3.2; to perform GLM and bootstrap was caret v6.0–86 and for the AUC analysis was the pROC library.

## Supplementary Information

**Additional file 1. Table S1**: Probability of binary responses for ALP and substrates' threshold levels vis-à-vis the interaction of time and genetic status.

**Additional file 2. Table S2**: List of subjects included in +GT group with heterozygous pathogenic or likely pathogenic ALPL variants.

## Data Availability

The datasets generated and/or analyzed during the current study are available from the corresponding author on reasonable request.
