# Supplementary material for: Biochemical algorithm to identify individuals with ALPL variants among subjects with persistent hypophosphatasaemia
Source: Orphanet J Rare Dis. 2022 Mar 3;17:98. doi: 10.1186/s13023-022-02253-5 (PMC8896389; doi:10.1186/s13023-022-02253-5)
Supplement: Supplementary file 1 — Additional file 1. Table S1: Probability of binary responses for ALP and substrates' threshold levels vis-à-vis the interaction of time and genetic status. [file 13023_2022_2253_MOESM1_ESM.docx]

**Supplementary Table 1. Probability of binary responses for ALP and substrates' threshold levels vis-à-vis the interaction of time and genetic status.**

|  | **Baseline** | | **1 year** | | **2 years** | |
| --- | --- | --- | --- | --- | --- | --- |
|  | **+GT** | **-GT** | **+GT** | **-GT** | **+GT** | **-GT** |
| **Prob. ALP<25, Mean (SE)** | 0.38 (0.09) | 0.16 (0.07) | 0.48 (0.1) | 0.09 (0.05) | 0.38 (0.1) | 0.08 (0.06) |
| **Prob. PLP>180, Mean (SE)** | 0.52 (0.09) | 0.03 (0.03) | 0.83 (0.07) | 0.17 (0.08) | 0.88 (0.07) | 0.10 (0.06) |
| **Prob. PEA >30, Mean (SE)** | 0.55 (0.09) | 0.18 (0.07) | 0.72 (0.09) | 0.17 (0.07) | 0.30 (0.09) | 0.14 (0.07) |

**Abbreviations:** +GT, positive genetic test group; -GT, negative genetic test group; Prob, probability of; SE, standard error; ALP, alkaline phosphatase (IU/L); PLP, serum pyridoxal-5'-phosphate (nmol/L); PEA, urinary phosphoetanolamine (µmol/g creatinine).
