# Supplementary material for: Biochemical algorithm to identify individuals with ALPL variants among subjects with persistent hypophosphatasaemia
Source: Orphanet J Rare Dis. 2022 Mar 3;17:98. doi: 10.1186/s13023-022-02253-5 (PMC8896389; doi:10.1186/s13023-022-02253-5)
Supplement: Supplementary file 2 — Additional file 2. Table S2: List of subjects included in +GT group with heterozygous pathogenic or likely pathogenic ALPL variants. [file 13023_2022_2253_MOESM2_ESM.docx]

**Supplementary Table 2. List of subjects included in +GT group with heterozygous pathogenic or likely pathogenic *ALPL* variants.**

| **ID** | **Clinical features** | **Variant (cDNA)**  **(NM_000478.6)** | **Variant**  **(amino acid)** | **Variant classifi-cation**  **(ACMG)** | **Ex/Int** | **Proband**  **or family member** |
| --- | --- | --- | --- | --- | --- | --- |
| 1 | YES | c.871G>A | p.(Glu291Lys) | P | Ex 9 | Proband |
| 2 | YES | c.334G>C | p.(Gly112Arg) | P | Ex 5 | Proband |
| 3 | YES | c.497C>T | p.(Thr166Ile) | P | Ex 8 | Proband |
| 4 | YES | c.871G>A | p.(Glu291Lys) | P | Ex 9 | Proband |
| 5 | YES | c.871G>A | p.(Glu291Lys) | P | Ex 9 | Proband |
| 6 | YES | c.1366G>A | p.(Gly456Arg) | P | Ex 12 | Proband |
| 7 | YES | c.343_348dup | p.(Thr115_Ala116dup) | P | Ex 5 | Proband |
| 8 | YES | c.343_348dup | p.(Thr115_Ala116dup) | P | Ex 5 | Family member |
| 9 | YES | c.382G>A | p.(Val128Met) | P | Ex 5 | Proband |
| 10 | YES | c.334G>C | p.(Gly112Arg) | P | Ex 5 | Proband |
| 11 | YES | c.1133A>G | p.(Asp378Gly) | P | Ex 10 | Proband |
| 12 | YES | c.659G>T | p.(Gly220Val) | P | Ex 12 | Proband |
| 13 | YES | c.1471G>A | p.(Gly491Arg) | LP | Ex 12 | Proband |
| 14 | YES | c.473-2A>G | p.(?) | P | Int 5 | Proband |
| 15 | YES | c.1283G>A | p.(Arg428Gln) | P | Ex 11 | Proband |
| 16 | YES | c.343_348dup | p.(Thr115_Ala116dup) | P | Ex 5 | Family member |
| 17 | YES | c.473-2A>G | p.(?) | P | Int 5 | Proband |
| 18 | YES | c.892G>A | p.(Glu298Lys) | P | Ex 9 | Proband |
| 19 | YES | c.343_348dup | p.(Thr115_Ala116dup) | P | Ex 5 | Proband |
| 20 | YES | c.407G>A | p.(Arg163His) | P | Ex 5 | Proband |
| 21 | YES | c.809G>A | p.(Trp270*) | P | Ex 8 | Proband |
| 22 | NO | c.454C>T | p.(Arg152Cys) | P | Ex 5 | Proband |
| 23 | YES | c.497C>T | p.(Thr166Ile) | LP | Ex 6 | Family member |
| 24 | YES | c.871G>T | p.(Glu291*) | P | Ex 9 | Proband |
| 25 | YES | c.571G>A | p.(Glu191Lys) | P | Ex 6 | Proband |
| 26 | YES | c.1471G>A | p.(Gly491Arg) | LP | Ex 12 | Proband |
| 27 | YES | c.388_389insG | p.(Val130Glyfs*6) | P | Ex 5 | Proband |
| 28 | YES | c.382G>A | p.(Val128Met) | P | Ex 5 | Proband |
| 29 | NO | c.547G>A | p.(Asp183Asn) | LP | Ex 6 | Proband |
| 30 | YES | c.619G>C | p.(Gln207Glu) | P | Ex 6 | Proband |
| 31 | YES | c.1426G>A | p.(Gly476Lys) | LP | Ex 5 | Proband |
| 32 | YES | c.1277G>A | p.(Gly426Asp) | P | Ex 11 | Proband |
| 33 | YES | c.334G>C | p.(Gly112Arg) | P | Ex 5 | Proband |
| 34 | YES | c.343_348dup | p.(Thr115_Ala116dup) | P | Ex 5 | Proband |
| 35 | YES | c.001T>A | p.(Val331Met) | P | Ex 9 | Proband |
| 36 | YES | c.1276G>A | p.(Gly426Ser) | P | Ex 11 | Proband |
| 37 | YES | c.407G>A | p.(Arg136His) | P | Ex 5 | Proband |
| 38 | YES | c.1120G>A | p.(Val374Met) | P | Ex 10 | Proband |
| 39 | YES | c.551G>A | p.(Arg184Gln) | P | Ex 6 | Proband |
| 40 | NO | c.1348C>T | p.(Arg450Cys) | P | Ex 12 | Proband |

**Abbreviations**: ALP, alkaline phosphatase (IU/L); Serum PLP, serum pyridoxalphosphate; Urinary PEA, urinary phosphoetanolamine; P, pathogenic; LP, likely pathogenic; Ex, exon; Int, intron.
